# Supplementary material for: Network-based disease fingerprinting with neuroinflammation PET imaging
Source: J Neuroinflammation. 2026 Apr 10;23:172. doi: 10.1186/s12974-026-03788-1 (PMC13214161; doi:10.1186/s12974-026-03788-1)
Supplement: Supplementary file 1 — Supplementary Material 1. [file 12974_2026_3788_MOESM1_ESM.pdf]

Network-based disease fingerprinting with neuroinflammation PET imaging

Leonardo Barzon, Lucia Maccioni, Michelle Carranza Mellana, Julia J. Schubert, Ludovica Brusaferri, Oliver Cousins, Ivana Rosenzweig, Yuya Mizuno, Tiago Reis Marques, Neil A. Harrison, Tim Fryer, Edward T. Bullmore, Valeria Mondelli, Carmine Pariante, David Sharp, Gregory Scott, Joana B. Pereira, Oliver Howes, Vesna Sossi, Benedetta Bodini, Bruno Stankoff, Marco L. Loggia, Federico E. Turkheimer, Mattia Veronese

Supplementary materials

S.1 Technical specifications of the PET scanners

Tracer	Site	Scanner model	Nominal resolution (FWHM)	FOV (axial × trans.) [cm]	Analysis resolution (voxel [mm])	TOF	Detector material	Reconstruction method	References
[^{11}C]-PBR28	KCL/ ICL	Siemens Biograph TruePoint PET-CT	~4.5 mm	21.6 × 70.0	2.051 × 2.051 × 2	No	LSO	FBP	(Bloomfield et al., 2016; Nair et al., 2016; Nettis et al., 2020; Scott et al., 2018; Veronese et al., 2018)
	MGH (Bay 6)	PET-MR: PET within Siemens 3T Tim Trio MR	~4.3 mm	19.2 × 60.0	1.25 × 1.25 × 1.25	No	LSO	OP-OSEM 3D	(Albrecht et al., 2019; Alshelh et al., 2020; Torrado-Carvajal et al., 2021)
	MGH (Bay 7)	Siemens Biograph mMR whole-body PET-MR	~4.4 mm	25.8 × 59.4	2.09 × 2.09 × 2.03	No	LSO	OP-OSEM 3D	(Morrissey et al., 2023)

	UBC	GE SIGNA PET-MR	~4.0 mm	25.0 × 60.0	1.39 × 1.39 × 2.78	Yes	LYSO	PSF-HYPR4D-K-TOF OSEM (Cheng et al., 2022)	n.a
**[^{18}F]-DPA714**	KCL	Siemens Biograph mMR PET-MR	~4.4 mm	25.8 × 59.4	1.402 × 1.402 × 2.031	No	LSO	FBP	(Cousins et al., 2023; Mizuno et al., 2025)
	ICM	GE SIGNA PET-MR	~4.0 mm	25.0 × 60.0	1.172 × 1.172 × 2.78	No	LYSO	OP-OSEM 3D	n.a.
	ICM	Siemens HRRT	~2.5 mm	25.5 × 31.2	1.219 × 1.219 × 1.219	No	LSO/LYSO	OP-OSEM 3D	(Hamzaoui et al., 2023)
**[^{11}C]-PK11195**	KCL	GE SIGNA PET-MR	~4.0 mm	25.0 × 60.0	2 × 2 × 2.78	Yes	LYSO	TOF OSEM	(Schubert et al., 2021; Turkheimer et al., 2021)

Table S1. Technical specifications of the PET scanners. This table summarizes the key technical parameters of the PET systems included in the study: scanner model, spatial resolution (full-width at half maximum, FWHM), axial and transaxial field of view (FOV), time-of-flight (TOF) capability, and scintillation detector material. Image reconstruction methodologies are specified as follows: FBP (filtered back projection), OP-OSEM (3D Ordinary Poisson Ordered-Subset Expectation Maximization), and TOF-OSEM (time-of-flight OSEM). References are provided for previous publications detailing site-specific acquisition and preprocessing protocols.

S.2 Image-derived input function (IDIF) and K_1 estimation

The image-derived input function (IDIF) was computed for the majority of dynamic PET scans using a semi-automated pipeline adapted from Maccioni et al. (2024), where a preliminary anatomical mask isolated the carotid siphons to segment the arterial blood signal via intensity thresholding of early dynamic frames. Only for *ICM* scans, segmentations of the internal carotids were already available from an established internal procedure. All these segmentations were further refined by correlation analysis of voxel-wise time-activity curves (TACs), selecting a subset of voxels with the highest peak amplitudes and inter-voxel correlation to represent the arterial input. The resulting IDIF was fitted with a tri-exponential model—applying linear regression to the ascending part and a sum of three exponentials to the descending part (Parsey et al., 2000)—to ensure a noise-free input for the kinetic analysis. As a minor methodological exception for a small subset of data (*UBC* cohort, n=21), the IDIF was instead extracted on site using a validated voxel-search method optimized for time-of-flight PET data (Cheng et al., 2022).

Regional tracer blood-to-brain influx rate constants (K_1) were then estimated using the 1T1K-IDIF framework, a single irreversible compartment model based on the assumption that, within a limited window after injection, tracer kinetics primarily reflect influx from blood to parenchyma with minimal interference from metabolites or venous efflux. Adherence to these modeling assumptions was originally ensured by optimizing the fitting time window for each tracer to maximize the correlation with arterial blood gold-standard K_1 estimates, as established in the validation studies by Maccioni et al. (2024) for [^{11}C]-PBR28 and [^{18}F]-DPA714. For [^{11}C]-PK11195, the fitting window was selected based on evidence demonstrating the method's reliability and internal consistency (Barzon et al., 2025). This optimization process yielded K_1 estimates with high statistical precision, as reflected by low coefficients of variation (CV) across all datasets, ensuring the robustness of the individual similarity matrices. Specifically, the optimized fitting windows were defined as 0-4 minutes for all [11C]-PBR28 scans; 0-5 minutes for [^{11}C]-PK11195 scans and [^{18}F]-DPA714 (*KCL*) scans; and 0–8 minutes for [^{18}F]-DPA714 (*ICM*) scans.

S.3 Distribution of inter-regional similarity at baseline and after TSPO blocking

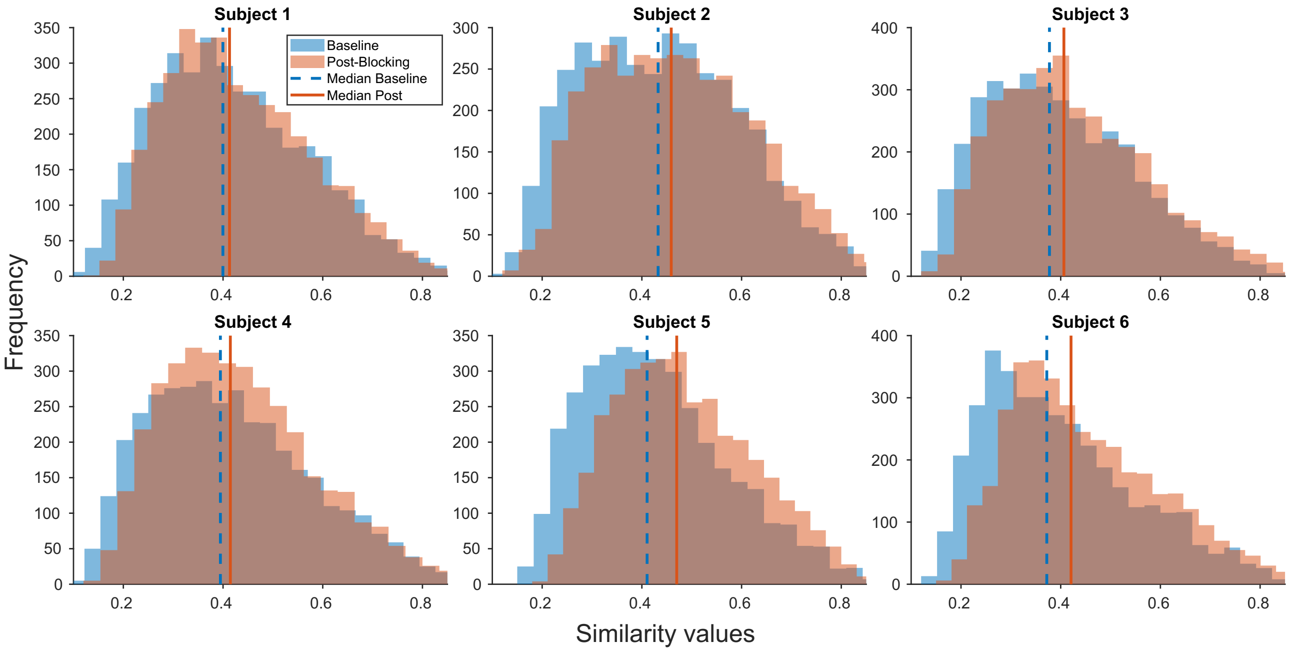

Figure S3. Histograms of pre- and post-blocking inter-regional similarity values. The plots illustrate the distributions of inter-regional similarity values for six schizophrenia (SCZ) subjects scanned with [^{11}C]-PBR28 at baseline and following partial pharmacological blocking of the TSPO target with XBD173 (Marques et al., 2021; Veronese et al., 2018). For each subject, baseline (blue) and post-blocking (red) distributions are overlaid to show the global shift in edge magnitude. The median of each distribution is highlighted.

S.4 Test-retest reliability and intraclass correlation coefficient matrices

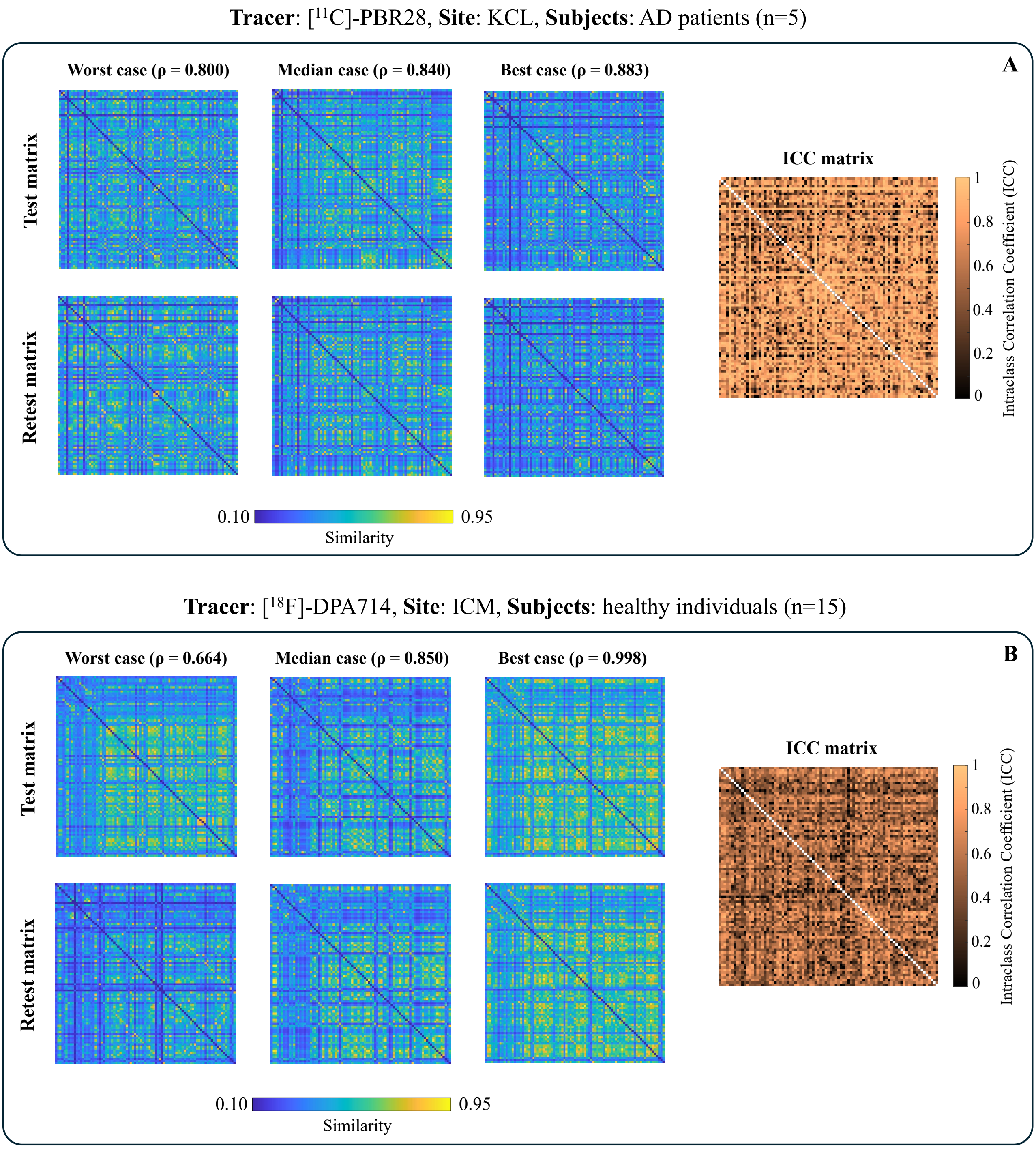

Figure S4. Test-retest reliability and intraclass correlation coefficient (ICC) matrices. Panels A and B illustrate the results for 5 AD patients (scanned at KCL with [11C]-PBR28) and 15 healthy controls (scanned at ICM with [18F]-DPA714), respectively. Within each panel, test and retest TSPO similarity matrices are displayed for the worst, median, and best-case examples of intrasubject Spearman correlation (ρ). Corresponding ρ values are reported above each pair. To the right of each panel, the matrices represent the edge-wise intraclass correlation coefficients (type ICC(3,1)) calculated across the cohort. Acronyms: AD, Alzheimer's disease; KCL, King's College London; ICM, Paris Brain Institute

S.5 Top network edges according to logistic regression coefficients for disease classification

**Traumatic brain injury**	**Multiple sclerosis**	**Chronic low back pain**
1. Lateral orbitofrontal area R ~ Lateral orbitofrontal area L (-0.15)<br>2. Medial orbitofrontal area L ~ Brainstem (0.13)<br>3. Rostral anterior cingulate area L ~ Putamen L (-0.11)<br>4. Medial orbitofrontal area L ~ Bankssts area L (-0.10)<br>5. Superior parietal area R ~ Paracentral area R (-0.09)	1. Putamen R ~ Caudate L (-0.06)<br>2. Rostral anterior cingulate area L ~ Accumbens area R (0.06)<br>3. Lingual area L ~ Cuneus area L (0.06)<br>4. Putamen R ~ Hippocampus L (-0.06)<br>5. Putamen L ~ Caudate L (-0.06)	1. Parahippocampal area L ~ Brainstem (-0.10)<br>2. Lateral occipital area R ~ Bankssts area L (-0.09)<br>3. Entorhinal area L ~ Ventral diencephalon L (0.09)<br>4. Temporal pole area R ~ Caudal anterior cingulate area R (0.09)<br>5. Rostral anterior cingulate area L ~ Precentral area L (0.08)
**Schizophrenia**	**Depression**	
1. Ventral diencephalon R ~ Amygdala L<br>2. (-0.10)<br>3. Precuneus area L ~ Putamen L (0.10)<br>4. Supramarginal area R ~ Caudal middle frontal area L (-0.10)<br>5. Pars triangularis area R ~ Rostral anterior cingulate area L (0.10)<br>6. Putamen R ~ Hippocampus L (-0.09)	1. Lateral occipital area L ~ Putamen R (0.20)<br>2. Temporal pole area R ~ Ventral diencephalon R (-0.16)<br>3. Pars opercularis area R ~ Rostral anterior cingulate area L (0.16)<br>4. Insula area R ~ Pars opercularis area L (0.15)<br>5. Pars triangularis area R ~ Pars orbitalis area R (-0.15)	

Figure S5. Top network edges for disease classification. For each disease-specific classifier, the figure shows the five edges with the largest absolute logistic regression coefficients. Positive coefficients (green) indicate higher similarity between the two regions in the patient group, whereas negative coefficients (red) indicate lower similarity in the disease group. Acronyms: L, left; R, right.

S.6 Multiclass disease classifier

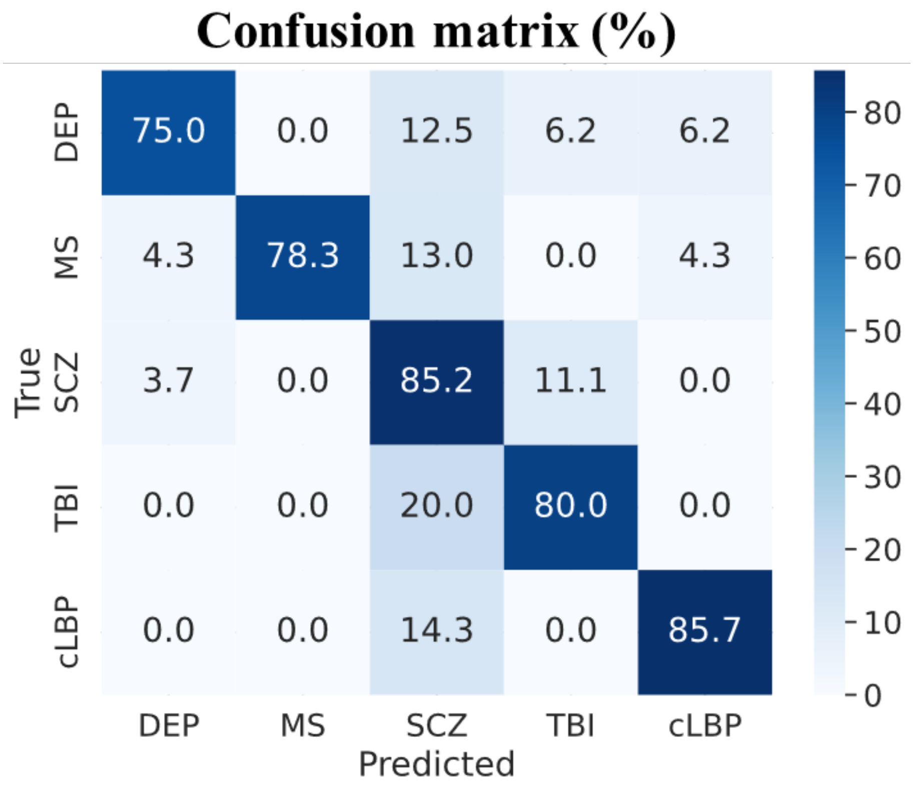

Figure S6. Multiclass disease classification performance. The figure illustrates the confusion matrix of test-set results from a multiclass logistic regression model. The model achieved a balanced accuracy [95% confidence interval] of 80.8 [67.6, 90.3] %, well above the 20% chance level. Acronyms: DEP, depression; MS, multiple sclerosis; SCZ, schizophrenia; TBI, traumatic brain injury; cLBP, chronic low back pain.

S.7 Site-specific classification models and comparison with corresponding multi-site models

			Classification performance		***Correlation of classifier β***	
**Group**	**Tracer**	**Site/dataset**	**AP [95% CI]**	**CL**	**Kendall τ**	**P**
SCZ	[^{11}C]-PBR28	KCL/ICL	0.79 [0.57, 0.96]	0.55	0.15	<0.001
SCZ	[^{11}C]-PBR28	UBC	0.87 [0.62, 1.00]	0.43	0.11	<0.001
FEP (SCZ)	[^{18}F]-DPA714	KCL	0.79 [0.65, 0.96]	0.61	0.37	<0.001
MS	[^{18}F]-DPA714	ICM (PET-MR)	0.91 [0.79, 0.99]	0.71	0.19	<0.001
MS	[^{18}F]-DPA714	ICM (HRRT)	0.92 [0.83, 0.99]	0.69	0.12	<0.001
cLBP	[^{11}C]-PBR28	MGH (Bay 6)	0.71 [0.50, 0.91]	0.50	0.09	<0.001

Table S7. Performance of site-specific classification models and comparison with corresponding multi-site models. The table reports the classification performance of the site-specific models and the Kendall's tau correlation between their β coefficients and those of the corresponding multi-site model. Acronyms: SCZ, schizophrenia; FEP, first-episode psychosis; MS, multiple sclerosis; cLBP, chronic low back pain; KCL, King's college London; ICL, Imperial College London; UBC, University of British Columbia; ICM, Paris Brain Institute (PET-MR and HRRT indicate the two scanners); MGH, Massachusetts General Hospital (Bay 6 indicates the scanner); AP, average precision; CL, average precision chance level.

References

Albrecht, D. S., Forsberg, A., Sandström, A., Bergan, C., Kadetoff, D., Protsenko, E., Lampa, J., Lee, Y. C., Höglund, C. O., Catana, C., Cervenka, S., Akeju, O., Lekander, M., Cohen, G., Halldin, C., Taylor, N., Kim, M., Hooker, J. M., Edwards, R. R., … Loggia, M. L. (2019). Brain glial activation in fibromyalgia – A multi-site positron emission tomography investigation. *Brain, Behavior, and Immunity*, *75*, 72–83. https://doi.org/10.1016/j.bbi.2018.09.018

Alshelh, Z., Albrecht, D. S., Bergan, C., Akeju, O., Clauw, D. J., Conboy, L., Edwards, R. R., Kim, M., Lee, Y. C., Protsenko, E., Napadow, V., Sullivan, K., & Loggia, M. L. (2020). In-vivo imaging of neuroinflammation in veterans with Gulf War illness. *Brain, Behavior, and Immunity*, *87*, 498–507. https://doi.org/10.1016/j.bbi.2020.01.020

Barzon, L., Maccioni, L., Moretto, M., Giacomel, A., Schubert, J. J., Cousins, O., Rosenzweig, I., Mizuno, Y., Marques, T. R., Harrison, N. A., Fryer, T., Bullmore, E. T., Mondelli, V., Pariante, C., Howes, O., Turkheimer, F. E., & Veronese, M. (2025). *Peripheral inflammation is associated with reduced influx of TSPO PET tracers into the brain: insights from a non-invasive mapping methodology*. https://doi.org/10.21203/rs.3.rs-6648321/v2

Bloomfield, P. S., Selvaraj, S., Veronese, M., Rizzo, G., Bertoldo, A., Owen, D. R., Bloomfield, M. A. P., Bonoldi, I., Kalk, N., Turkheimer, F., McGuire, P., de Paola, V., & Howes, O. D. (2016). Microglial Activity in People at Ultra High Risk of Psychosis and in Schizophrenia: An [^{11} C]PBR28 PET Brain Imaging Study. *American Journal of Psychiatry*, *173*(1), 44–52. https://doi.org/10.1176/appi.ajp.2015.14101358

Cheng, J.-C. K., Bevington, C. W. J., & Sossi, V. (2022). HYPR4D kernel method on TOF PET data with validations including image-derived input function. *EJNMMI Physics*, *9*(1), 78. https://doi.org/10.1186/s40658-022-00507-6

Cousins, O., Schubert, J. J., Chandra, A., Veronese, M., Valkimadi, P., Creese, B., Khan, Z., Arathimos, R., Hampshire, A., Rosenzweig, I., Ballard, C., Corbett, A., Aasland, D., Velayudhan, L., O'Neill, M., Collier, D., Awais, R., Sander, K., Årstad, E., … Hodges, A. (2023). Microglial activation, tau and amyloid deposition in TREM2 p.R47H carriers and mild cognitive impairment patients: a multi-modal/multi-tracer PET/MRI imaging study with influenza vaccine immune challenge. *Journal of Neuroinflammation*, *20*(1). https://doi.org/10.1186/s12974-023-02945-0

Hamzaoui, M., Garcia, J., Boffa, G., Lazzarotto, A., Absinta, M., Ricigliano, V. A. G., Soulier, T., Tonietto, M., Gervais, P., Bissery, A., Louapre, C., Bottlaender, M., Bodini, B., & Stankoff, B. (2023). Positron Emission Tomography with [18F]-DPA-714 Unveils a Smoldering Component in Most Multiple Sclerosis Lesions which Drives Disease Progression. *Annals of Neurology*, *94*(2), 366–383. https://doi.org/10.1002/ana.26657

Maccioni, L., Michelle, C. M., Brusaferri, L., Silvestri, E., Bertoldo, A., Schubert, J. J., Nettis, M. A., Mondelli, V., Howes, O., Turkheimer, F. E., Bottlaender, M., Bodini, B., Stankoff, B., Loggia, M. L., & Veronese, M. (2024). A blood-free modeling approach for the quantification of the blood-to-brain tracer exchange in TSPO PET imaging. *Frontiers in Neuroscience*, *18*, 1395769. https://doi.org/10.3389/fnins.2024.1395769

Marques, T. R., Veronese, M., Owen, D. R., Rabiner, E. A., Searle, G. E., & Howes, O. D. (2021). Specific and non-specific binding of a tracer for the translocator-specific protein in schizophrenia: an [11C]-PBR28 blocking study. *European Journal of Nuclear Medicine and Molecular Imaging*, *48*(11), 3530–3539. https://doi.org/10.1007/s00259-021-05327-x

Mizuno, Y., Carreira Figueiredo, I., Pillinger, T., Hindley, G., Baxter, L., Parmar, S., Lobo, M. C., Donocik, J. G., Rosenzweig, I., Gupta, A., Callegari, I., Jeljeli, S., Dunn, J. T., Hammers, A., Awais, R., Sander, K., Årstad, E., Politis, M., Schubert, J. J., … Howes, O. D. (2025). Immune alterations in schizophrenia and the effects of a therapeutic antibody: a neuroimaging study. *Brain*. https://doi.org/10.1093/brain/awaf455

Morrissey, E. J., Alshelh, Z., Knight, P. C., Saha, A., Kim, M., Torrado-Carvajal, A., Zhang, Y., Edwards, R. R., Pike, C., Locascio, J. J., Napadow, V., & Loggia, M. L. (2023). Assessing the potential anti-neuroinflammatory effect of minocycline in chronic low back pain: Protocol for a randomized, double-blind, placebo-controlled trial. *Contemporary Clinical Trials*, *126*, 107087. https://doi.org/10.1016/j.cct.2023.107087

Nair, A., Veronese, M., Xu, X., Curtis, C., Turkheimer, F., Howard, R., & Reeves, S. (2016). Test-retest analysis of a non-invasive method of quantifying [11C]-PBR28 binding in Alzheimer's disease. *EJNMMI Research*, *6*(1), 72. https://doi.org/10.1186/s13550-016-0226-3

Nettis, M. A., Veronese, M., Nikkheslat, N., Mariani, N., Lombardo, G., Sforzini, L., Enache, D., Harrison, N. A., Turkheimer, F. E., Mondelli, V., & Pariante, C. M. (2020). PET imaging shows no changes in TSPO brain density after IFN-α immune challenge in healthy human volunteers. *Translational Psychiatry*, *10*(1). https://doi.org/10.1038/s41398-020-0768-z

Parsey, R. V., Slifstein, M., Hwang, D. R., Abi-Dargham, A., Simpson, N., Mawlawi, O., Guo, N. N., Van Heertum, R., John Mann, J., & Laruelle, M. (2000). Validation and reproducibility of measurement of 5-HT(1A) receptor parameters with [carbonyl-11 C]WAY-100635 in humans: Comparison of arterial and reference tissue input functions. *Journal of Cerebral Blood Flow and Metabolism*, *20*(7). https://doi.org/10.1097/00004647-200007000-00011

Schubert, J. J., Veronese, M., Fryer, T. D., Manavaki, R., Kitzbichler, M. G., Nettis, M. A., Mondelli, V., Pariante, C. M., Bullmore, E. T., Turkheimer, F. E., Wlazly, D., Dickinson, A., Foster, A., Knight, C., Leckey, C., Morgan, P., Morgan, A., O'Hagan, C., Touchard, S., … Mount, H. (2021). A Modest Increase in 11C-PK11195-Positron Emission Tomography TSPO Binding in Depression Is Not Associated With Serum C-Reactive Protein or Body Mass Index. *Biological Psychiatry: Cognitive Neuroscience and Neuroimaging*, *6*(7), 716–724. https://doi.org/10.1016/j.bpsc.2020.12.017

Scott, G., Zetterberg, H., Jolly, A., Cole, J. H., De Simoni, S., Jenkins, P. O., Feeney, C., Owen, D. R., Lingford-Hughes, A., Howes, O., Patel, M. C., Goldstone, A. P., Gunn, R. N., Blennow, K., Matthews, P. M., & Sharp, D. J. (2018). Minocycline reduces chronic microglial activation after brain trauma but increases neurodegeneration. *Brain*, *141*(2), 459–471. https://doi.org/10.1093/brain/awx339

Torrado-Carvajal, A., Toschi, N., Albrecht, D. S., Chang, K., Akeju, O., Kim, M., Edwards, R. R., Zhang, Y., Hooker, J. M., Duggento, A., Kalpathy-Cramer, J., Napadow, V., & Loggia, M. L. (2021). Thalamic neuroinflammation as a reproducible and discriminating signature for chronic low back pain. *Pain*, *162*(4), 1241–1249. https://doi.org/10.1097/j.pain.0000000000002108

Turkheimer, F. E., Althubaity, N., Schubert, J., Nettis, M. A., Cousins, O., Dima, D., Mondelli, V., Bullmore, E. T., Pariante, C., & Veronese, M. (2021). Increased serum peripheral C-reactive protein is associated with reduced brain barriers permeability of TSPO radioligands in healthy volunteers and depressed patients: implications for inflammation and depression. *Brain, Behavior, and Immunity*, *91*, 487–497. https://doi.org/10.1016/j.bbi.2020.10.025

Veronese, M., Reis Marques, T., Bloomfield, P. S., Rizzo, G., Singh, N., Jones, D., Agushi, E., Mosses, D., Bertoldo, A., Howes, O., Roncaroli, F., & Turkheimer, F. E. (2018). Kinetic modelling of [11C]PBR28 for 18 kDa translocator protein PET data: A validation study of vascular modelling in the brain using XBD173 and tissue analysis. *Journal of Cerebral Blood Flow & Metabolism*, *38*(7), 1227–1242. https://doi.org/10.1177/0271678X17712388
